# Establishing diagnostic criteria for feline obesity using a highly sensitive serum amyloid A assay

**DOI:** 10.3389/fvets.2025.1630963

**Published:** 2025-07-03

**Authors:** Miki Kobayashi, Motoo Kobayashi, Tomoaki Hirose, Masashi Yuki, Satoru Imano, Yukari Asahi, Takahiro Teshima, Toshiro Arai

**Affiliations:** ^1^Seijo Kobayashi Veterinary Clinic, Tokyo, Japan; ^2^Yuki Animal Hospital, Nagoya, Japan; ^3^Eiken Chemical Co., Ltd., Tokyo, Japan; ^4^Nippon Veterinary and Life Science University, Musashino, Japan

**Keywords:** ALT, cat, obesity disease, SAA, triglycerides

## Abstract

**Introduction:**

The global increase in the prevalence of overweight and obesity is associated with several chronic diseases. Obesity is characterized by systemic low-grade inflammation and oxidative stress caused by excessive fat accumulation. This study aimed to establish diagnostic criteria for pathological obesity in cats using a highly sensitive serum amyloid A (SAA) assay.

**Methods:**

In this study, 29 client-owned cats (3–14 years old) with varying body condition scores (BCS) were included. These cats underwent medical checkups and were not treated for any specific diseases. They were divided into three groups: healthy control, simple obesity, and obesity disease. The plasma levels of metabolites, hormones, and enzymes were measured.

**Results:**

In the simple obesity cats, body weight, BCS, and plasma triglyceride (TG) and malondialdehyde (MDA) concentrations were significantly higher than those in the healthy control cats. In the obesity disease cats, BCS was significantly higher than that in the simple obesity cats, and plasma TG and SAA concentrations and alanine aminotransferase (ALT) activities increased significantly compared to those in the simple obesity cats. Adiponectin concentrations in the obesity disease cats were significantly lower than those in the healthy control cats.

**Discussion:**

The novel criteria for feline obesity (overweight cats exhibiting two or more of the following symptoms: hyperlipidemia and high ALT and SAA levels) were based on biomarker values that were significantly higher than those in the simple obesity cats. These novel criteria may help detect pathological obesity at an early stage. Early and accurate diagnosis can prevent age-related diseases including obesity.

## 1 Introduction

The global increase in the prevalence of overweight and obesity is associated with several chronic diseases (1). Obesity is characterized by systemic low-grade inflammation and oxidative stress caused by excessive fat accumulation. Obesity superimposed on aging represents an additional risk factor for older age groups, in which the prevalence of chronic diseases and occurrence of complications increases (2, 3). Obesity is associated with numerous comorbidities, including hypertension, type 2 diabetes mellitus, dyslipidemia, obstructive sleep apnea and sleep-disordered breathing, certain types of cancers, and major cardiovascular diseases (4). In cats, whose glucose and lipid metabolism differs from that of dogs, obesity and its associated diseases increase significantly with age (5, 6). In general, the prevalence of obesity in cats is assumed to be 30–40% (7, 8), but it is even higher, >50% in the USA (9) and 63% in New Zealand (10). Obesity is classified into two types: with and without health issues (5). In human medicine, obesity accompanied by health issues is defined as obesity disease (pathological obesity) (11, 12). Cats are more prone to be obese than dogs (13), and the classification of obesity is crucial for diagnosing its early stages to prevent various obesity-associated diseases. We have previously established similar criteria for feline obesity disease (6). However, these criteria are less common in veterinary medicine owing to their inconvenient use. Recently, a highly sensitive serum amyloid A (SAA) assay was developed (14), capable of detecting low concentrations of SAA that serves as a diagnostic marker for chronic inflammation in cats.

In this study, we measured plasma metabolite and hormone concentrations and enzyme activities in healthy and obese cats to develop diagnostic criteria for obesity. These criteria may be useful for detecting early stages of obesity to suppress age-related diseases in cats.

## 2 Materials and methods

### 2.1 Animals

This study included 29 clinically healthy client-owned cats (3–14 years old) with varying body condition scores (BCS) from two veterinary hospitals in Tokyo, Japan. The cats underwent checkups and were not treated for any specific disease (Table 1). Written informed consent was obtained from all the owners. Twenty-nine cats were divided into three groups according to their BCS and plasma triglyceride (TG) concentrations. Group 1 (*n* = 12) BCS ≤5, TG <180 mg/100 mL; Group 2 (*n* = 12) BCS 5–7, TG <180 mg/100 mL; Group 3 (*n* = 5) BCS >7, TG ≥180 mg/100 mL.

**Table 1:** Characteristics of the cats examined in this study.

No.	Breeds	Sex	Age (years)	BW (kg)	BCS
Group 1 (BCS ≤5, TG <180 mg/100 mL)					
1	Mongrel	NM	4.4	4.4	5/9 points
2	British Shorthair	NM	10.5	3.8	5
3	Singapura	NF	4.5	2.4	5
4	Mongrel	NM	13.3	5.2	5
5	Mongrel	NM	10	4.2	5
6	Munchkin	NF	14.5	3.4	5
7	Mongrel	NF	10.9	2.9	4
8	Mongrel	NM	4.7	5.3	5
9	American Shorthair	Male	6.1	4.6	5
10	Mongrel	NM	6.8	3.7	5
11	Munchkin	NF	8.8	3.4	5
12	Munchkin	NM	11.2	4.8	5
Group 2 (BCS 5–7, TG <180 mg/100 mL)					
13	Mongrel	NF	7.7	5.8	6
14	Mongrel	NF	7.2	4.8	6
15	Somali	Male	3.3	7.2	6
16	Mongrel	NM	14.1	6.7	6
17	Ragamuffin	NF	3.0	5.0	6
18	American Shorthair	NM	11.5	8.3	7
19	Mongrel	NF	12.2	4.7	6
20	Mongrel	Male	9.6	6.8	7
21	Scottish Fold	NM	8.1	5.6	6
22	Mongrel	NM	5.8	5.5	6
23	Mongrel	NM	6.8	6.3	7
24	Mongrel	NF	11.5	4.6	6
Group 3 (BCS >7, TG ≥180 mg/100 mL)					
25	Munchkin	NF	9.0	6.1	8
26	Mongrel	NF	6.5	6.8	7
27	Mongrel	NM	8.6	7.9	9
28	Mongrel	Male	9.6	6.7	8
29	Russian Blue	Male	5.3	5.9	7

BW, body weight; BCS, body condition score; TG, triglyceride; NM, neutered male; NF, neutered female.

### 2.2 Blood sampling and body weight and BCS measurement

Preprandial blood was collected from the jugular vein, and plasma was separated by centrifugation using heparin. Plasma was stored at −80°C. Body weight (BW) and BCS were measured during blood sampling. BCS was assessed using a 9-point scale system (15), where 1 indicated emaciation, 5 was ideal, and 9 indicated extreme fat.

### 2.3 Plasma metabolite, hormone, and enzyme assay

Plasma concentrations of glucose, triglyceride, total cholesterol, total protein, creatinine, blood urea nitrogen (BUN), and aspartate aminotransferase (AST) and alanine aminotransferase (ALT) activities were measured using an autoanalyzer (DRY-CHEM NX7000; FUJIFILM Corporation, Tokyo, Japan), following the manufacturer’s protocols. Plasma free fatty acid (FFA) concentrations were measured using a commercial kit (NEFA-C Test; Wako Pure Chemical Industries Ltd., Tokyo, Japan). Plasma SAA concentrations were measured using the veterinary SAA (VET-SAA) kit (Eiken Chemical Co., Tokyo, Japan). Plasma malondialdehyde (MDA) and adiponectin concentrations were measured using the NWLSSTM Malondialdehyde assay kit (Northwest Life Science Specialties, LLC, Vancouver, Canada) and LBIS^™^ Mouse/Rat High Molecular Weight Adiponectin ELISA kit (FUJIFILM Corporation, Tokyo, Japan), respectively.

### 2.4 Statistical analysis

Measured values are expressed as the mean ± standard error. Statistical significance was determined using Mann–Whitney *U*-test. The significance level was set at *p* < 0.05.

## 3 Results

Table 2 presents the plasma metabolite and hormone concentrations and enzyme activities in the three groups of cats (*n* = 27). Cats 2 and 5 in Group 1 exhibited abnormally high ALT (>80 U/L) and MDA (>60 μmol/L) levels compared to those of the other cats and were excluded from the study. Cats 23 and 24 in Group 2 were diagnosed as obesity disease based on the previous diagnostic criteria for feline obesity disease (6), and moved to the obesity disease group. The cats were divided into three groups—healthy control (*n* = 10), simple obesity (*n* = 10), and obesity disease (*n* = 7).

**Table 2:** Alterations in plasma biomarker levels in the healthy and obesity disease cats.

	Healthy control (10)	Simple obesity (10)	Obesity disease (7)
Body weight (kg)	3.9 ± 0.3	6.0 ± 0.4^a^	6.3 ± 0.4^a^
Body condition score	4.9 ± 0.1	6.1 ± 0.2^a^	7.4 ± 0.3^a^^,^^b^
Glucose (mg/100 mL)	117 ± 8	116 ± 8	148 ± 9^a^
Triglyceride (mg/100 mL)	52 ± 8	86 ± 9^a^	400 ± 113^a^^,^^b^
Total cholesterol (mg/100 mL)	156 ± 18	199 ± 17	185 ± 16
Free fatty acid (mEq/L)	0.49 ± 0.05	0.40 ± 0.06	0.89 ± 0.21^a^^,^^b^
Total protein (g/100 mL)	7.4 ± 0.2	7.4 ± 0.2	7.3 ± 0.3
Creatinine (mg/100 mL)	1.3 ± 0.1	1.3 ± 0.1	1.4 ± 0.0
BUN (mg/100 mL)	27 ± 2	27 ± 2	25 ± 1
AST (U/L)	37 ± 3	25 ± 2^a^	35 ± 3
ALT (U/L)	54 ± 3	51 ± 3	89 ± 6^a^^,^^b^
Serum amyloid A (mg/L)	1.2 ± 0.2	1.4 ± 0.5	4.1 ± 0.7^a^^,^^b^
Malondialdehyde (μmol/L)	8 ± 2	22 ± 4^a^	56 ± 17^a^
Adiponectin (μg/mL)	3.0 ± 0.4	1.9 ± 0.2^a^	1.4 ± 0.1^a^

Values are presented as the mean ± standard error. The number in parentheses is the number of animals examined. BUN, blood urea nitrogen; AST, aspartate aminotransferase; ALT, alanine aminotransferase.

a Significantly different from healthy control cat values (*p* < 0.05).

b Significantly different from simple obesity cat values (*p* < 0.05).

In the simple obesity cats, BW, BCS, and plasma TG and MDA concentrations were significantly higher than those in the healthy control cats. BCS in the obesity disease cats increased significantly than that in the simple obesity cats. In the obesity disease cats, plasma TG, FFA and SAA concentrations and ALT activities increased significantly compared to those in the simple obesity cats. Adiponectin concentrations in the obesity diseases cats were significantly lower than those in the healthy control cats.

Feline obesity disease was diagnosed if overweight cats with BCS ≥7/9 demonstrate two or more of the following symptoms—hyperlipidemia (TG ≥180 mg/100 mL), high ALT activities (≥80 U/L), and high SAA concentrations (≥4.0 mg/L) (Table 3).

**Table 3:** Diagnostic criteria of obesity disease in cats.

Overweight (BCS ≥7/9) With two or more of the following symptoms Hyperlipidemia (TG ≥180 mg/100 mL) High ALT (≥80 U/L) High SAA (≥4.0 mg/L)

BCS, body condition score; TG, triglyceride; ALT, alanine aminotransferase; SAA, serum amyloid A.

## 4 Discussion

Obesity is a significant public health concern worldwide, and its prevalence in cats has increased with age, similar to that observed in humans (7, 16). Age-related diseases, including obesity, are accelerated by a chronic low-grade proinflammatory state known as inflammaging (17). There are no specific medicines for age-related diseases, and early diagnosis and intervention are effective in preventing disease progression. Measuring inflammatory markers is crucial for treating obesity in animals. SAA is a well-known acute-phase reaction that is elevated in acute inflammatory conditions, such as infection, tissue injury, and trauma (18). Differences in SAA expression kinetics between acute inflammation and chronic disease states indicate distinct functional roles for the SAA. Although circulating SAA levels can increase by up to 1,000-fold during an acute inflammatory event, the increase is modest (approximately 5-fold) in chronic metabolic conditions (19). A system for measuring low SAA levels in chronic inflammatory conditions is necessary for diagnosing feline obesity. Currently, SAA concentrations in cats are measured in many studies (20, 21) using an automated turbidimetric assay (latex turbidimetric immunoassay for SAA [LZ-SAA]) based on a mixture of anti-human SAA-specific monoclonal and polyclonal antibodies. In this study, VET-SAA—that uses an animal-specific monoclonal antibody, exhibited higher sensitivity than that of LZ-SAA against feline SAA and can accurately detect low levels of SAA (<5 mg/L) (14, 22). Yuki et al. (14) clarified that the sensitivity and specificity of VET-SAA in diagnosing disease cats (cutoff, 4.0 mg/L) using Receiver operating characteristic (ROC) analysis with over 200 cats. SAA ≥4.0 mg/L was set as cutoff value for diagnosing low-grade inflammation in the obesity disease cats (Table 3).

Prominent differences in obesity disease cats from simple obesity cats were dyslipidemia, ectopic fat accumulation and systemic chronic low-grade inflammation. Dyslipidemia, ectopic fat accumulation and chronic low-grade inflammation were reflected as high TG concentrations, high ALT activities and high SAA concentrations, respectively. Hyperglycemia with plasma TG levels >400 mg/100 mL was observed in the obesity disease cats, and these values were significantly higher than those in the simple obesity cats. However, plasma total cholesterol concentrations were not as high as those in the simple obesity cats. Fatty liver is more likely to occur, and hepatic lipid metabolism is affected in obese cats (6, 7, 23, 24). Various biomarkers are used to assess fatty liver disease in obese humans (25–27). Elevated ALT activities were used to assess nonalcoholic fatty liver disease (NAFLD) compared with biopsies, ultrasound scans, or magnetic resonance imaging (28). Additionally, it has been assessed as a sensitive biomarker to diagnose fatty liver disease (27, 28). In this study, ALT activities in the obesity disease cats were significantly higher than those in the simple obesity cats, with elevated ALT activities reflecting the severity of hepatic injury. TG ≥180 mg/100 mL and ALT ≥80 U/L were set as cutoff values for diagnosing obesity disease based on results in the present study and our previous studies with over 180 obese cats (8, 29).

Chronic inflammatory conditions tend to cause a much lower elevation in systemic SAA (approximately 5 to 10-food) than that in acute inflammatory conditions and may be present in various tissues, such as the liver, adipose tissue, lungs, small and large intestines, and hematopoietic cells, such as macrophages (30, 31). Obesity generates a self-feeding cycle of monocyte/macrophage infiltration to sustain low-grade chronic inflammation of white adipose tissue, and expanding adipocytes themselves produce various mediators such as SAA, ILs, TNF-α and MCP-1 (32). Elevated SAA concentrations (approximately 3.5-fold of the control) in the obesity disease cats were likely derived from accumulated visceral fat and fatty liver.

Reduced adiponectin levels were included in the previous diagnostic criteria for feline obesity. However, measuring adiponectin concentrations is less common in veterinary medicine. In this study, plasma adiponectin concentrations were reduced in the obesity disease cats, but the reduction was not significantly different from that in the simple obese cats. Therefore, we excluded low adiponectin concentrations from the novel diagnostic criteria for feline obesity.

Our novel criteria for feline obesity (overweight cats exhibiting two or more of the following symptoms: hyperlipidemia and high ALT activities and SAA concentrations) were based on biomarker values that were significantly higher than those in the simple obesity cats. These novel criteria may help detect pathological obesity at an early stage. Early and accurate diagnosis can prevent age-related diseases, including obesity, through supplementation with phytochemicals, such as resveratrol (22) and quercetin (33). Although our preliminary criteria demonstrate promise, their application in routine clinical practice requires further validation in larger and more diverse feline populations, ideally through multicentric and longitudinal studies, before being adopted as diagnostic standards. Additionally, it would be valuable to conduct longitudinal follow-up of cohorts with simple versus pathological obesity, including periodic measurements of the proposed biomarkers (especially SAA), to assess their dynamics over time and validate their utility in monitoring disease progression.

The limitations of this study include the small sample size and biological and environmental variables (age, sex, neuter status, gestation, diet, cage feeding, in door or out door, and activity levels etc.) that are inevitable when using client-owned animals from two veterinary hospitals. Because castration contributes to the development of obesity in male cats (34, 35), further studies involving animals of various ages, sexes, and districts are necessary to assess the obesity criteria. Data measured using VET-SAA in cats with chronic inflammatory conditions are limited. Although our biomarker-based criteria provide a practical tool for early diagnosis of feline pathological obesity, their robustness would be strengthened by complementary imaging techniques. Future studies should incorporate hepatic ultrasound to assess steatosis and fibrosis, as well as dual-energy X-ray absorptiometry (DEXA) to quantify the distribution of visceral versus subcutaneous fat. These data would allow for direct correlation of TG, ALT, and SAA levels with the extent and localization of lipid accumulation, thereby improving threshold validation and facilitating clinical translation of this protocol. To strengthen the validity of the proposed ALT (≥80 U/L) and TG (≥180 mg/100 mL) thresholds, it would be beneficial to complement serum biomarker data with imaging techniques and, where possible, histological evaluation. Future studies incorporating hepatic ultrasound to assess steatosis and fibrosis, or DEXA/CT scans to quantify visceral versus subcutaneous fat, would help correlate plasma biomarkers with fat distribution. In selected cases, liver biopsy samples could provide histopathological confirmation of liver damage indicated by elevated ALT activities, thus enhancing the diagnostic robustness of the proposed criteria.

## 5 Conclusion

Clinically healthy client-owned cats (3–14 years old) with varying BCS were included in the study and divided into three groups: healthy control, simple obesity, and obesity disease. In the simple obesity cats, BW, BCS, and plasma TG and MDA concentrations increased significantly than those in the healthy control cats. In the obesity disease cats, plasma TG, FFA and SAA concentrations and ALT activities increased significantly compared to those in the simple obesity cats. Adiponectin concentrations in the obesity diseases cats were significantly lower than those in the healthy control cats. Feline obesity disease was diagnosed if overweight cats with BCS ≥7/9 demonstrated two or more of the following symptoms—hyperlipidemia (TG ≥180 mg/100 mL), high ALT activities (≥80 U/L), and high SAA concentrations (≥4.0 mg/L). The novel criteria for feline obesity were based on biomarker values that were significantly higher than those in the simple obesity cats. These novel criteria may help detect obesity disease (pathological obesity) at an early stage. Early and accurate diagnosis can prevent age-related diseases, including obesity in cats.

## Data Availability

The raw data supporting the conclusions of this article will be made available by the authors, without undue reservation.
